# Blood Flow Restriction Training for Rehabilitation and Readiness in Tactical Populations: A Scoping Review

**DOI:** 10.3390/healthcare14182920

**Published:** 2026-09-09

**Authors:** Haonan Tian, Aozhe Wang, Lin Yan, Longhao Xiao, Jun Wang

**Affiliations:** 1 School of Sport Science, Beijing Sport University, Beijing 100084, China; 2 School of Physical Education, Anhui Polytechnic University, Wuhu 241000, China; 3 National Olympic Sports Center, Beijing 100029, China; 4 Laboratory of Sports Stress and Adaptation of General Administration of Sport, Beijing Sport University, Beijing 100084, China

**Keywords:** blood flow restriction training, tactical populations, musculoskeletal rehabilitation, occupational health, return to duty, physical fitness

## Abstract

**Highlights:**

**What are the main findings?**

- Completed BFRT studies in tactical populations have increased in recent years, but all eligible studies involved military personnel, with none identified in other tactical occupations
- The evidence is dominated by small, short-term studies of rehabilitation and physical fitness, with limited assessment of occupational performance, safety, and implementation.

**What are the implications of the main findings?**

- Current evidence is insufficient to support routine BFRT implementation across tactical populations, and direct evidence regarding occupational readiness remains limited.
- Larger and longer studies should broaden population representation and prioritize task-specific, return-to-duty, safety, and implementation outcomes with more standardized BFRT reporting.

**Abstract:**

**Background/Objectives**: Tactical populations must maintain high physical capacity and operational readiness despite substantial occupational demands and musculoskeletal injury risk. Blood flow restriction training (BFRT) has potential applications in injury rehabilitation and the restoration or maintenance of physical capacity. However, BFRT research in tactical populations has not been systematically mapped. This review aimed to characterize the existing evidence. **Methods**: This scoping review followed JBI methodology and PRISMA-ScR guidance. Seven databases and two clinical trial registries were searched. Two reviewers independently conducted study screening and data extraction. Evidence was summarized descriptively and graphically. **Results**: Thirty reports representing 21 studies were included, comprising 17 completed studies and 4 protocol- or registry-only studies. All completed studies involved military populations, and all planned studies targeted military populations. No eligible studies involving other tactical occupations were identified. Completed studies generally had small BFRT-exposed samples and short intervention durations, with male representation more common than female representation across studies. They mainly examined rehabilitation and fitness/performance. Individualized occlusion-pressure prescription and low-load resistance exercise were common. Strength/power was the most frequently assessed observed outcome, whereas occupational performance was rarely assessed. Safety and implementation reporting in completed studies remained incomplete. **Conclusions**: Completed BFRT studies in tactical populations have increased in recent years, but the evidence remains limited to military populations and exploratory in nature. Future studies should include non-military tactical occupations and more women, use larger samples and longer interventions, assess occupationally relevant outcomes, and improve the completeness and consistency of BFRT prescription, implementation, and safety reporting.

## 1. Introduction

Although occupational groups such as military personnel, law enforcement officers, firefighters, and emergency medical responders differ in their specific task demands, operational environments, and injury patterns, they are commonly considered within the broader framework of “tactical populations” in the literature [1,2]. This is because these occupations share several characteristics, including the need to perform prolonged and physically demanding duties in complex, unpredictable, and potentially hazardous environments while maintaining a high level of operational readiness. Physical attributes such as muscular strength, power, aerobic capacity, and anaerobic capacity are essential for maintaining occupational performance, personnel safety, and operational readiness [3]. To preserve these physical capabilities, tactical populations typically undertake systematic and continuous physical training. However, repeated training stimuli and high occupational demands also place this population at considerable risk of musculoskeletal injury. Musculoskeletal injuries are a major cause of training interruption, work restrictions, and reduced operational readiness [4,5]. Therefore, although the specific task demands of different tactical occupations should not be considered equivalent, maintaining or restoring physical capacity while controlling mechanical load represents a broadly relevant issue in tactical physical training and injury rehabilitation. In this review, readiness refers to the capacity to perform or resume occupational duties. We distinguish direct readiness outcomes, such as return to duty and performance on occupation-specific or task-simulation tests, from indirect or surrogate outcomes, such as muscular strength, power, pain, aerobic or anaerobic capacity, and general physical function. Although these indirect or surrogate outcomes may contribute to occupational readiness, favorable changes in them alone should not be interpreted as direct evidence of improved readiness without corresponding occupation-specific outcomes.

Blood flow restriction training (BFRT) is an exercise method in which external pressure is applied to the proximal portion of a limb to restrict venous return while partially limiting arterial inflow during exercise [6]. BFRT can be combined with low-load resistance training, aerobic exercise, and other forms of training [7,8,9]. Compared with traditional high-load training, BFRT can induce substantial local metabolic and neuromuscular stimuli at relatively low external loads, thereby promoting relevant physiological adaptations [10]. Previous systematic reviews and meta-analyses have suggested that BFRT may improve muscular adaptations and selected physical performance outcomes in healthy adults and competitive athletes [11,12]. Positive effects have also been reported in clinical rehabilitation settings, including rehabilitation following anterior cruciate ligament reconstruction and the management of non-traumatic knee osteoarthritis and related knee conditions [13,14,15].

These characteristics provide a theoretical rationale for the application of BFRT in tactical populations. In healthy tactical personnel, BFRT may serve as an adjunct to traditional tactical physical training and may help maintain an effective training stimulus when external mechanical loading needs to be reduced or when access to training equipment is limited [16]. During post-injury or postoperative rehabilitation, BFRT may allow personnel who cannot tolerate higher loads to begin progressive strength training at an earlier stage and may provide a transition toward the resumption of traditional high-load training [17]. However, tactical populations are exposed to distinct occupational demands, training loads, and operational environments [3]. Therefore, findings from general or clinical populations may not be directly generalizable to these populations. Importantly, the practical applicability of BFRT depends not only on its potential physiological or clinical effects, but also on whether it can be delivered safely and consistently. Screening for contraindications, individualized cuff-pressure determination, monitoring of exercise-related discomfort and adverse events, and appropriate supervision are important considerations for its implementation. In addition, although some BFRT devices are relatively portable [18], portability alone does not establish feasibility during deployment, field training, or other resource-limited settings, where equipment reliability, personnel requirements, and protocol implementation may also influence its practical use.

In recent years, the application of BFRT in tactical populations has received increasing attention, although the available evidence remains fragmented. To our knowledge, only one previous review has specifically addressed BFRT in tactical populations. That narrative review focused on military personnel with persistent pain following complex injuries and primarily discussed the rationale for BFRT, potential analgesic mechanisms, and its clinical application in pain management [17]. However, that review did not aim to systematically map BFRT applications beyond pain management or across the broader range of tactical occupations. Therefore, the present scoping review extends the existing literature by systematically mapping BFRT research within a broader tactical-population framework, with particular attention to the populations and application contexts studied, BFRT prescription and outcome characteristics, and evidence gaps related to readiness, safety, and implementation. Given that scoping reviews are well suited to mapping the breadth of evidence, research characteristics, and knowledge gaps in emerging fields [19], this approach was considered appropriate for the present review. Specifically, this scoping review addressed three questions. First, which tactical populations and application contexts have been studied using BFRT, and what are the characteristics of these studies? Second, how are BFRT prescriptions and major outcome categories distributed across the existing literature? Third, how have safety and implementation domains, including adverse events and discomfort, feasibility, adherence, acceptability, supervision, implementation fidelity, and protocol deviations, been reported, and what evidence gaps remain?

## 2. Methods

This scoping review followed the Joanna Briggs Institute (JBI) methodology for scoping reviews [20] and was reported in accordance with the Preferred Reporting Items for Systematic Reviews and Meta-Analyses extension for Scoping Reviews (PRISMA-ScR) guidelines [21]. The completed PRISMA-ScR checklist is provided in Supplementary Table S1.

The original review protocol was finalized on 1 May 2026, before the formal literature search was conducted on 20 May 2026. Title and abstract screening was conducted from 21 May to 23 May 2026, full-text screening from 24 May to 29 May 2026, and data extraction from 5 June to 26 June 2026. The protocol was retrospectively registered on the Open Science Framework (OSF) on 11 August 2026, and is available at https://osf.io/n3ykw (accessed on 6 September 2026). The original review protocol is provided as Supplementary File S1. No substantive deviations from the original protocol occurred. As prespecified, the data extraction form was piloted before formal extraction. During this planned piloting process, several prespecified domains were further operationalized into more specific data extraction categories to improve the consistency and completeness of data charting. These refinements did not alter the eligibility criteria, review questions, or principal domains specified in the original protocol.

### 2.1. Information Sources and Search Strategy

To identify studies examining the application of BFRT in tactical populations, we systematically searched PubMed, Embase, Scopus, Web of Science, SPORTDiscus (EBSCO), CINAHL (EBSCO), and the Cochrane Central Register of Controlled Trials (CENTRAL). To identify ongoing or unpublished studies, we also searched ClinicalTrials.gov and the ISRCTN Registry. Searches covered the period from database inception to 20 May 2026.

Search terms were determined through discussion among all authors and adapted to the subject headings, free-text terms, field tags, and search syntax of each database. The search terms primarily included terms related to BFRT and tactical populations. The overall search strategy was: (“blood flow restriction” OR “blood-flow restriction” OR “blood flow restriction training” OR “blood flow restriction exercise” OR “blood flow restriction therapy” OR “BFR” OR “BFRT” OR “occlusion training” OR “vascular occlusion training” OR “KAATSU” OR “KAATSU training”) AND (“military” OR “soldier*” OR “servicemember*” OR “service member*” OR “warfighter*” OR “armed force*” OR “army” OR “navy” OR “naval” OR “marine*” OR “air force” OR “cadet*” OR “ROTC” OR “special force*” OR “special operation*” OR “tactical athlete*” OR “tactical population*” OR “tactical personnel” OR “tactical operator*” OR “police” OR “law enforcement” OR “firefighter*” OR “fire fighter*” OR “first responder*” OR “emergency responder*” OR “paramedic*” OR “emergency medical technician*” OR “EMT”). The complete Boolean search strategies for all databases are provided in Supplementary Table S2.

### 2.2. Eligibility Criteria

The inclusion and exclusion criteria were developed according to the Population–Concept–Context (PCC) framework to define the target population, core concept, and application context of this scoping review. The specific inclusion and exclusion criteria are presented in Table 1.

Given that this review aimed to comprehensively map the distribution of evidence in this field, in addition to published original studies, conference abstracts, study protocols, and clinical trial registry records that were relevant to the review topic and provided sufficient extractable information were also included. Narrative reviews, systematic reviews, meta-analyses, editorials, and commentaries were excluded. For studies that included both tactical and non-tactical populations, the study was included only when data relating specifically to the tactical population could be extracted separately.

No language restrictions were applied during the literature search or screening process. Articles published in languages other than Chinese or English were independently translated with the assistance of two machine-translation tools, DeepSeek-V4-Pro (DeepSeek, Hangzhou, China) and DeepL Translator (DeepL SE, Cologne, Germany). Two researchers (H.T. and A.W.) independently reviewed both translated versions, with particular attention to information relevant to eligibility assessment and key data extraction, and cross-checked these details between the two translations. Any discrepancies or semantic ambiguities between the translated versions were jointly reviewed by the two researchers and resolved through discussion within the research team.

### 2.3. Study Screening and Eligibility Assessment

All records identified through the bibliographic database or trial registry searches were imported into Zotero reference management software (version 9.0.4; Corporation for Digital Scholarship, Vienna, VA, USA) for management. After duplicate records were removed, two researchers (H.T. and A.W.) independently screened titles and abstracts against the eligibility criteria to identify potentially relevant studies. The full texts of potentially eligible records were then independently assessed by the same two researchers. When the information provided in the title and abstract was insufficient to determine eligibility, the record was retained for full-text assessment.

For eligible clinical trial registry records identified through the database and registry searches, related publications were traced using the registration number, study title, investigator names, and intervention-related keywords to identify full-text reports arising from the same registered study. When a registry record corresponded to a published article or results report, the registry record and associated publications were treated as multiple reports of the same study and were not counted as separate studies. If no corresponding publication or results report could be identified, the registry record was described as a separate registered study. Published articles were not systematically traced in the reverse direction to identify corresponding trial registrations.

Any disagreements between the two researchers during the screening process were resolved through discussion. If consensus could not be reached, a third researcher (J.W.) was consulted for adjudication. The study selection process was reported using a PRISMA flow diagram.

### 2.4. Data Extraction

In accordance with the JBI guidance for data extraction in scoping reviews [22], an initial electronic data extraction form was developed. Before formal data extraction, two researchers (H.T. and A.W.) piloted the form using a subset of the included studies. The form was then revised based on the pilot extraction to ensure that the data collected adequately addressed the research questions and captured the target population, core concept, and context of the review. Formal data extraction was performed independently by two researchers (H.T. and A.W.), followed by cross-checking of the extracted data. Any disagreements arising during data extraction were resolved through discussion. If consensus could not be reached, a third researcher (J.W.) was consulted for adjudication.

Extracted variables included study and participant characteristics, BFRT and exercise prescription characteristics, application focus, outcome domains, and safety and implementation characteristics. Categorical variables were coded using standardized operational definitions and coding rules that operationalized the prespecified extraction domains and were refined during the planned piloting of the data extraction form. The complete operational definitions, category boundaries, and coding rules are provided in the “Coding Dictionary” worksheet of Supplementary Table S3.

During data extraction and coding, reports containing observed empirical data, including conference abstracts reporting empirical results, were distinguished from protocols or registry records without available results. When both planned and completed reports were available for the same study, observed data were extracted and coded from the completed empirical report. For protocol- or registry-only studies without available results, information on planned sample sizes, intervention procedures, outcomes, and safety and implementation characteristics was coded separately from observed data in completed studies. Information explicitly described as intended or planned was assigned a “planned” status, whereas variables not specified in the protocol or registry record were coded as “not specified.” These categories were distinguished from “not reported/unclear,” which was used for missing or insufficiently reported information in completed empirical studies.

### 2.5. Data Synthesis and Presentation

Data were organized, categorized, and summarized using Microsoft Excel (version 16.111.2; Microsoft Corporation, Redmond, WA, USA). Results were presented using descriptive statistics and narrative synthesis. To improve the clarity and readability of the results, visualizations were created using R (version 4.3.3; R Foundation for Statistical Computing, Vienna, Austria) and BioRender (https://www.biorender.com, accessed on 6 September 2026). The raw data extracted for this review are provided in the “Data” worksheet of Supplementary Table S3. Completed empirical studies and protocol- or registry-only studies were distinguished throughout the descriptive synthesis and separately labeled in Supplementary Table S3 and the relevant visualizations.

In accordance with the purpose and methodological framework of scoping reviews [21,22], no methodological quality assessment or risk-of-bias assessment was conducted for the included studies, and no meta-analysis was performed. Accordingly, the results of this review primarily mapped and described the scope of BFRT applications, study characteristics, and distribution of evidence across tactical populations, rather than estimate the pooled effects of BFRT as an intervention or formulating clinical recommendations.

## 3. Results

### 3.1. Study Selection

The database and trial registry searches identified a total of 350 records. After removal of 204 duplicates, 146 records underwent title and abstract screening. Following this screening, 102 records were excluded, and 44 reports were sought for full-text retrieval. Two reports could not be retrieved, leaving 42 reports for full-text eligibility assessment. After full-text assessment, 14 reports were excluded: eight involved non-tactical populations, five had unconfirmed tactical occupational status, and one did not involve BFRT or training. In addition, by tracing eligible clinical trial registry records, two further relevant full-text reports [23,24] were identified, both of which met the inclusion criteria. In total, 30 reports were included in the data extraction.

**Figure 1:**
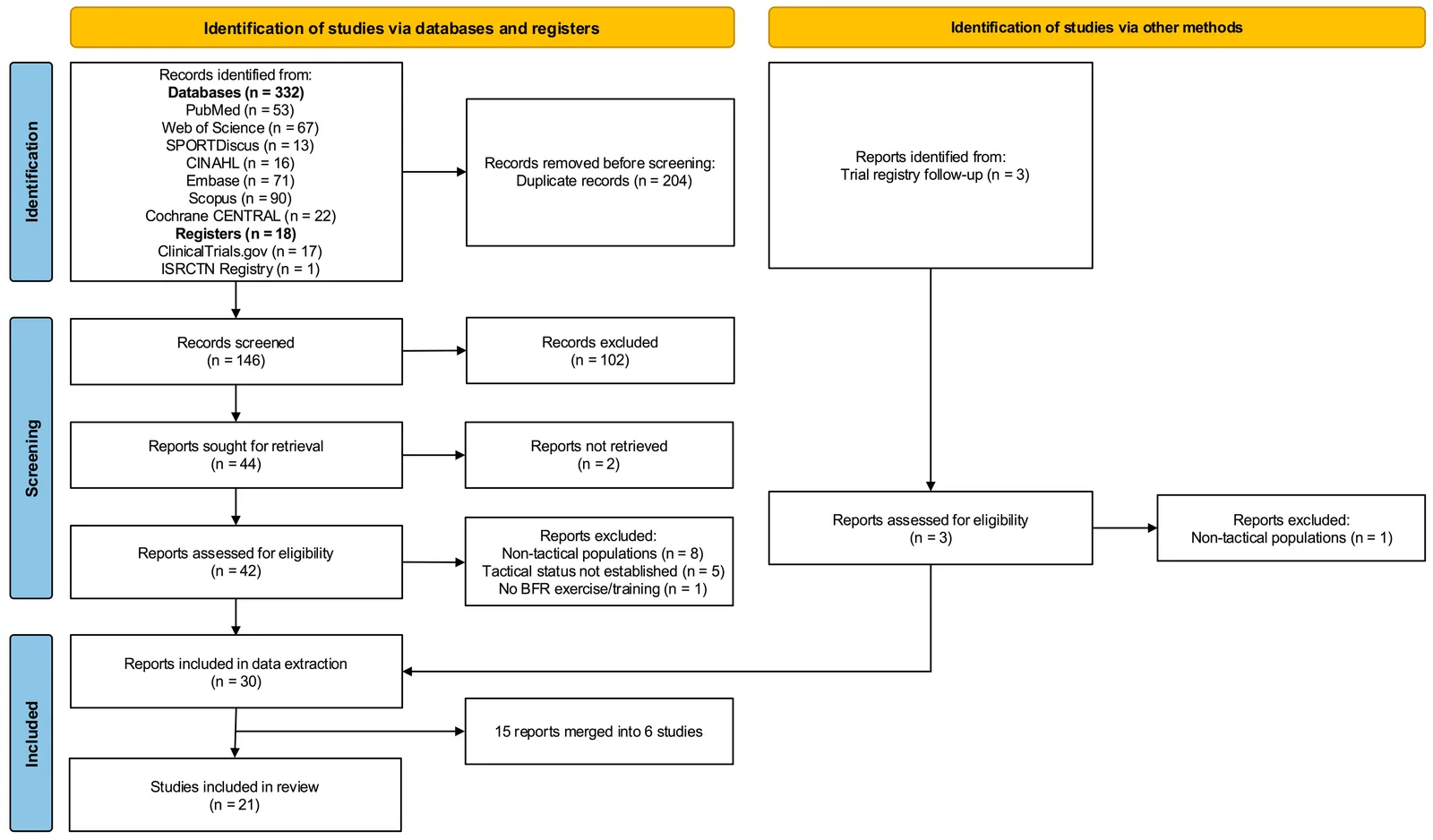
PRISMA flow diagram of study selection.

As some studies were represented by multiple reports, reports from the same study were grouped to avoid duplicate counting of the same data items in this scoping review [22]. Specifically, 15 reports [16,23,24,25,26,27,28,29,30,31,32,33,34,35,36] were consolidated into six studies, while the remaining 15 reports [37,38,39,40,41,42,43,44,45,46,47,48,49,50,51] each represented a separate study. Therefore, a total of 21 independent studies were included in this scoping review (Figure 1). The correspondence between all included reports and the independent studies is provided in the “Study–Report Linkage” worksheet of Supplementary Table S3.

### 3.2. Publication Trends, Study Design, and BFRT-Exposed Sample Size

The earliest completed study was published in 2016. Between 2016 and 2021, seven of the 17 completed studies (41.2%) were published, whereas 10 (58.8%) were published in 2022 or later. In addition, all four protocol- or registry-only studies were published or registered in 2022 or later. Geographically, the completed studies were primarily conducted in the United States and the United Kingdom, including eight studies from the United States, three from the United Kingdom, three from Brazil, two from Iran, and one from China. Among the four protocol- or registry-only studies, three were from the United Kingdom and one was from the United States.

**Figure 2:**
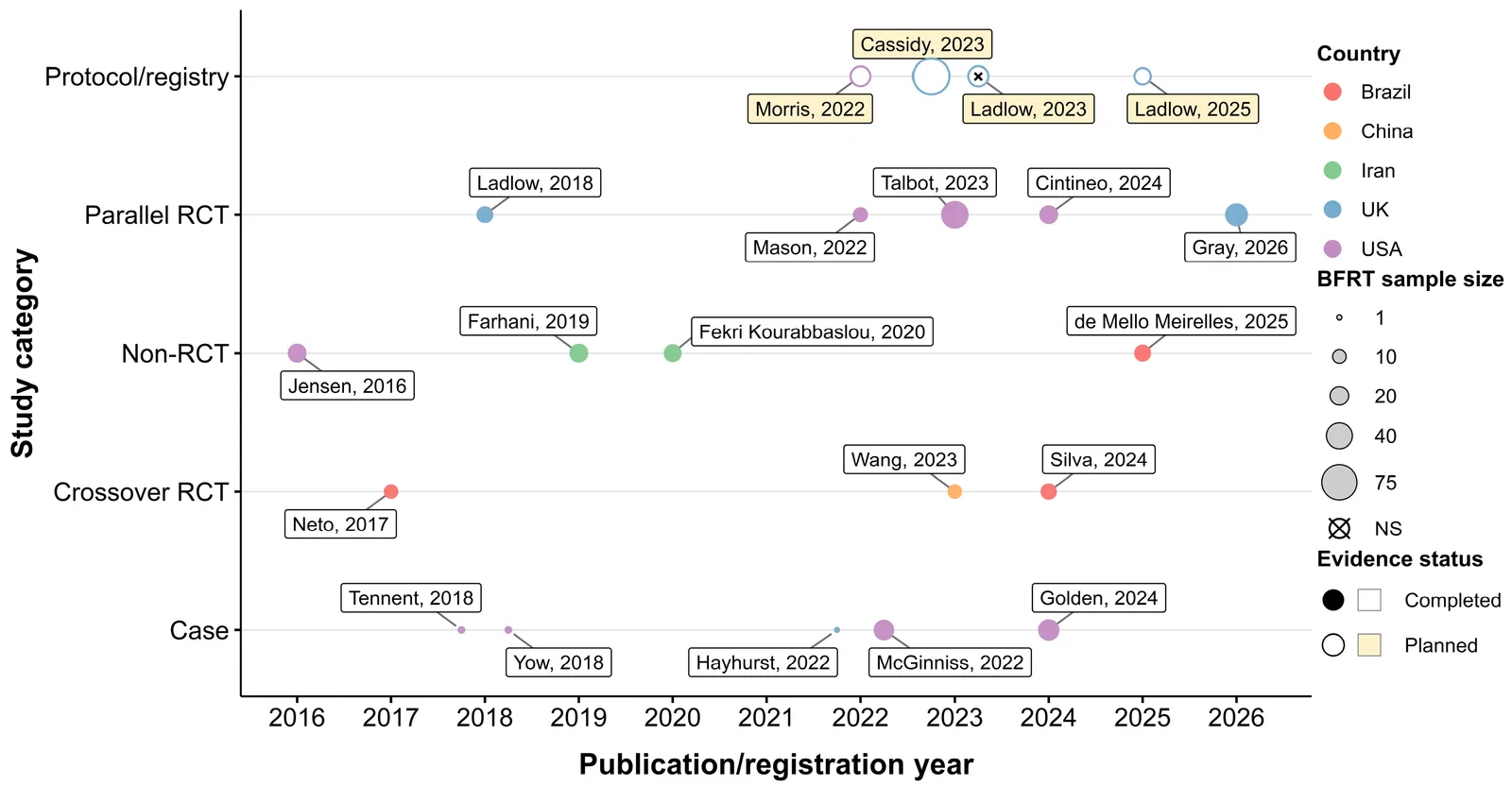
Distribution of included studies by publication or registration year, country, study category, BFRT-exposed sample size, and evidence status. Bubble color indicates country. Bubble size represents the actual number of participants exposed to BFRT in completed empirical studies and the planned number of participants intended to receive BFRT in protocol- or registry-only studies. Filled circles denote completed empirical studies, whereas open circles denote protocol- or registry-only studies; label backgrounds are also used to distinguish completed from planned evidence. NS indicates that the planned BFRT sample size was not specified. Parallel RCT, parallel-group randomized controlled trial; Non-RCT, non-randomized intervention study; Crossover RCT, randomized crossover trial; Case, case report or case series. Study labels correspond to the following references: Jensen, 2016 [41]; Neto, 2017 [47]; Ladlow, 2018 [25,33,34]; Tennent, 2018 [49]; Yow, 2018 [51]; Farhani, 2019 [38]; Fekri Kourabbaslou, 2020 [23,24,32]; Hayhurst, 2022 [40]; Mason, 2022 [42]; McGinniss, 2022 [43]; Morris, 2022 [44]; Cassidy, 2023 [26,27]; Ladlow, 2023 [45]; Talbot, 2023 [35,36]; Wang, 2023 [50]; Cintineo, 2024 [16,28]; Golden, 2024 [39]; Silva, 2024 [48]; Ladlow, 2025 [46]; de Mello Meirelles, 2025 [37]; and Gray, 2026 [29,30,31].

The 17 completed studies comprised five parallel-group randomized controlled trials, four non-randomized intervention studies, three randomized crossover trials, and five case reports or case series. All 17 completed studies reported actual BFRT-exposed sample sizes, with a median of 14 participants and a range of 1 to 42. Thirteen studies had an actual BFRT-exposed sample size of no more than 20 participants, representing 76.5% of the completed studies (Figure 2).

### 3.3. Participant Characteristics

All 17 completed studies involved military populations, and no completed studies involving other eligible tactical occupational groups, such as firefighters, law enforcement personnel, or emergency medical personnel, were identified. Among the completed studies, nine (52.9%) did not specify a military branch and described participants only as military personnel or service members. The remaining studies involved Army personnel (4/17, 23.5%), Navy personnel (3/17, 17.6%), Air Force personnel (3/17, 17.6%), special operations personnel (1/17, 5.9%), and military cadets (3/17, 17.6%). These military subgroup categories were not mutually exclusive because some studies included participants from more than one branch or category. Regarding sex, 15 of the 17 completed studies (88.2%) included male participants and six (35.3%) included female participants, while two studies (11.8%) did not clearly report participant sex. Only one study reported sex-stratified results [16]. In terms of health status, eight completed studies (47.1%) involved healthy participants and nine (52.9%) involved clinical or symptomatic populations.

**Figure 3:**
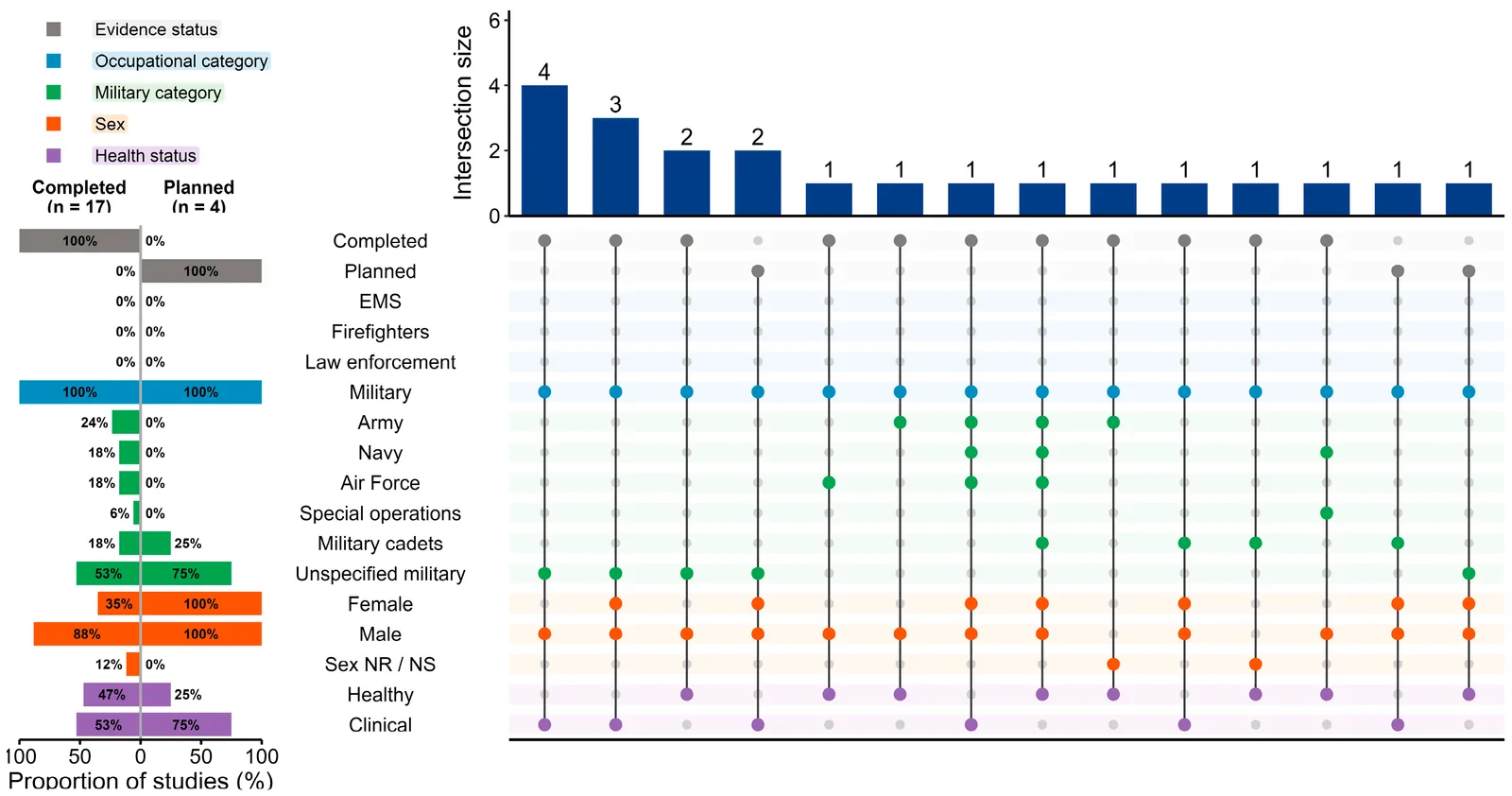
Distribution and intersections of participant characteristics by evidence status. The left panel shows the proportion of completed empirical studies and protocol- or registry-only studies with the corresponding occupational categories, military subgroups, sex categories, and health status. The right panel shows study-level intersections of these characteristics, with the bars above indicating the number of studies in each combination. For protocol- or registry-only studies, participant characteristics represent the intended target population specified in the protocol or registry record rather than observed characteristics of enrolled participants. Sex categories indicate the proportion of studies including at least one female or male participant and do not represent the number or proportion of individual participants by sex. NR indicates not reported or unclear in completed empirical studies; NS indicates not specified in protocol- or registry-only studies.

The four protocol- or registry-only studies were considered separately because their participant characteristics represented intended eligibility or target populations rather than observed enrollment. All four planned studies targeted military populations; three (75.0%) did not specify a military branch, whereas one (25.0%) involved military cadets. All four specified eligibility for both female and male participants. One planned study (25.0%) targeted healthy participants and three (75.0%) targeted clinical or symptomatic populations. No protocol- or registry-only studies involving non-military tactical occupations were identified (Figure 3).

### 3.4. Clinical Sites, BFRT Target Body Regions, and Limb Involvement

**Figure 4:**
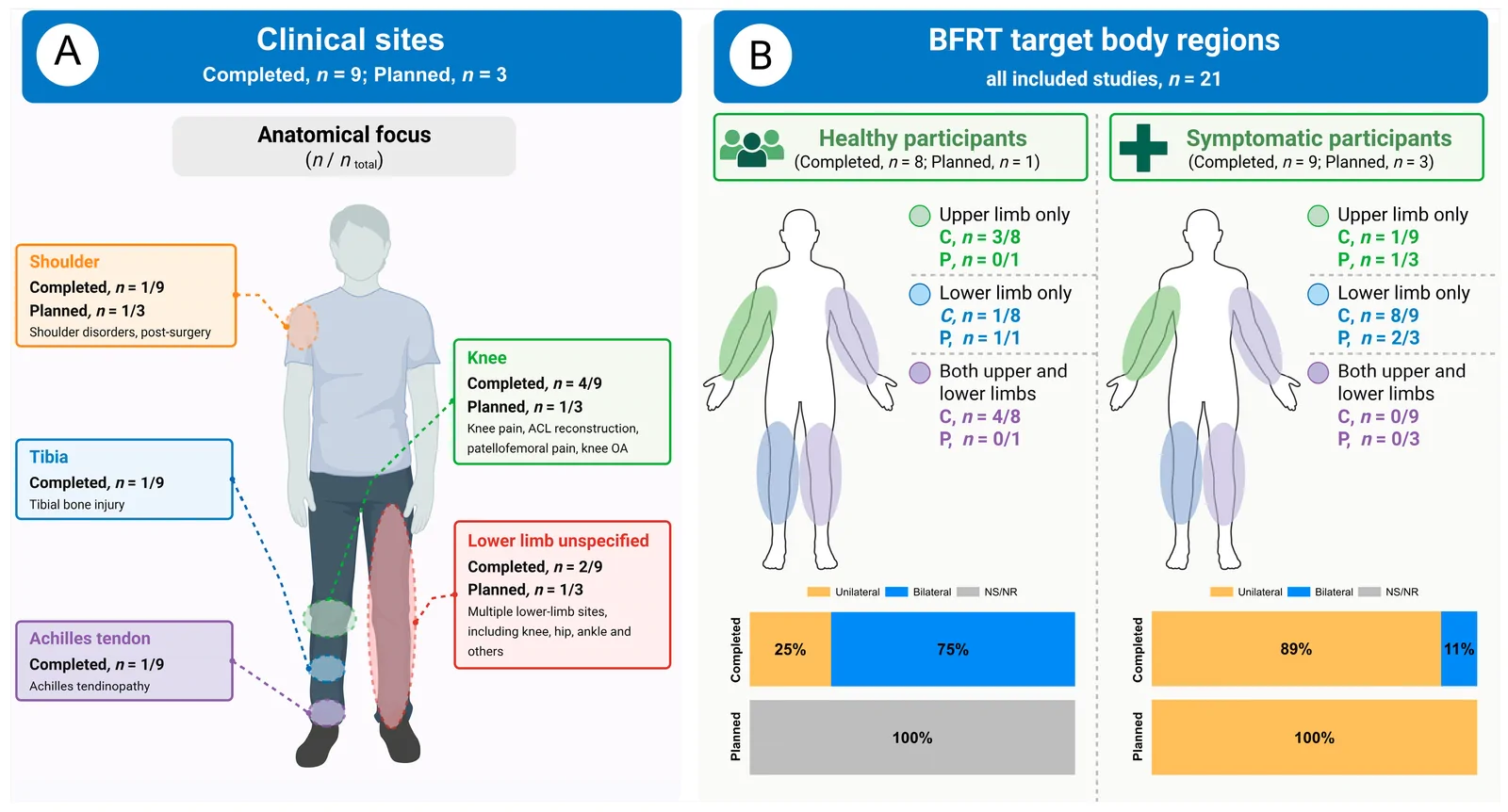
Clinical sites, BFRT target body regions, and limb involvement by evidence status. (**A**) Distribution of clinical sites among symptomatic participants. (**B**) Distribution of BFRT target body regions and limb laterality among healthy and symptomatic participants. For protocol- or registry-only studies, clinical sites represent the intended target population specified in the protocol or registry record, whereas BFRT target body regions and limb involvement represent planned intervention procedures rather than observed intervention delivery. C, completed empirical studies; P, planned studies (protocol- or registry-only studies); NR, not reported or unclear in completed empirical studies; NS, not specified in protocol- or registry-only studies. Created with BioRender.com.

Among the nine completed studies involving symptomatic participants, clinical conditions predominantly involved lower-limb musculoskeletal disorders or injuries. Knee-related conditions were the most common (4/9 studies), while tibial and Achilles tendon conditions were each reported in one study. Two additional studies reported lower-limb injuries without further specifying the anatomical site. Only one completed study involved a shoulder condition. Among the three protocol- or registry-only studies involving symptomatic populations, the planned clinical populations included one study of a shoulder condition, one of a knee condition, and one of a lower-limb condition without a clearly specified anatomical site (Figure 4A).

BFRT target body regions and limb laterality differed across participant groups. Among completed studies involving healthy participants, BFRT was applied to the upper limbs only in 3/8 studies, the lower limbs only in 1/8, and both upper and lower limbs in 4/8. Bilateral application was more common than unilateral application (75% vs. 25%). Among completed studies involving symptomatic participants, BFRT was predominantly applied to the lower limbs (8/9 studies) and was usually unilateral (89%). Among protocol- or registry-only studies, one study involving healthy participants planned lower-limb BFRT, whereas among the three studies involving symptomatic participants, one planned upper-limb BFRT and two planned lower-limb BFRT. All three planned studies involving symptomatic participants specified unilateral application, whereas limb laterality was not specified in the planned study involving healthy participants (Figure 4B).

### 3.5. BFRT Parameter Characteristics

**Figure 5:**
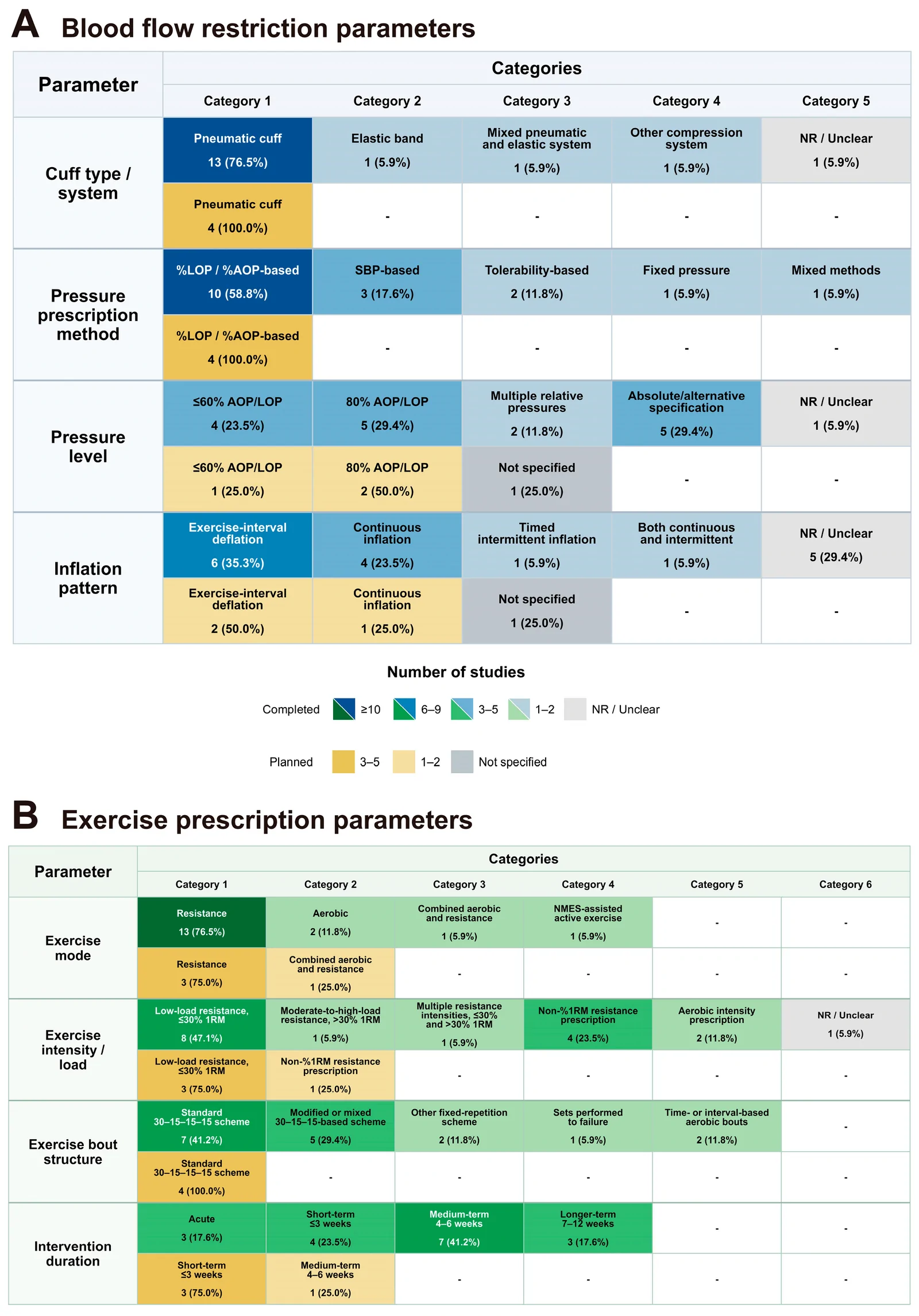
BFRT and exercise prescription characteristics by evidence status. (**A**) Distribution of blood flow restriction parameters. (**B**) Distribution of exercise prescription parameters. Counts and percentages were calculated separately for completed studies (*n* = 17) and protocol- or registry-only studies (*n* = 4). For protocol- or registry-only studies, the presented parameters represent intended intervention procedures specified in the protocol or registry record rather than observed intervention delivery. NR/Unclear indicates information that was not reported or could not be determined in completed empirical studies, whereas Not specified indicates information not specified in protocol- or registry-only studies. LOP, limb occlusion pressure; AOP, arterial occlusion pressure; SBP, systolic blood pressure; 1RM, one-repetition maximum; NMES, neuromuscular electrical stimulation; NR, not reported.

Among the 17 completed studies, pneumatic cuffs were the most commonly used blood flow restriction device (13/17, 76.5%), and cuff pressure was most frequently prescribed relative to individual limb occlusion pressure (LOP) or arterial occlusion pressure (AOP) (10/17, 58.8%). The actual applied pressure levels varied across studies, with five studies using 80% AOP/LOP, four using ≤60% AOP/LOP, and five using absolute pressure or other pressure specifications. Cuff inflation and deflation patterns also varied: six studies deflated the cuff during exercise intervals, four maintained continuous inflation throughout the exercise session, and five did not clearly report the inflation–deflation pattern (Figure 5A). Regarding exercise prescription, resistance exercise was the predominant modality (13/17, 76.5%), with low-load resistance exercise at ≤30% of one-repetition maximum (1RM) representing the most common intensity category (8/17, 47.1%). Seven studies used the standard 30–15–15–15 repetition scheme, whereas five used a modified 30–15–15-based scheme. Actual intervention durations in the completed empirical studies ranged from a single acute session to 7–12 weeks, with most lasting no longer than 6 weeks (14/17, 82.4%) (Figure 5B).

Among the four protocol- or registry-only studies, all planned to use pneumatic cuffs and to prescribe cuff pressure individually on the basis of LOP/AOP. Planned pressure levels included ≤60% AOP/LOP in one study and 80% AOP/LOP in two studies, whereas one study did not specify the planned pressure level. Two studies planned cuff deflation during exercise intervals, one planned continuous inflation throughout the exercise session, and one did not specify the planned inflation–deflation pattern. Regarding planned exercise prescription, three studies planned to use resistance exercise alone, whereas one planned a combined aerobic and resistance exercise modality. Three studies planned low-load resistance exercise at ≤30% 1RM, while one used a non-%1RM resistance prescription. All four studies planned to use the standard 30–15–15–15 repetition scheme. Planned intervention duration was ≤3 weeks in three studies and 4–6 weeks in one study (Figure 5).

### 3.6. Application Focus and Outcome Domains

**Figure 6:**
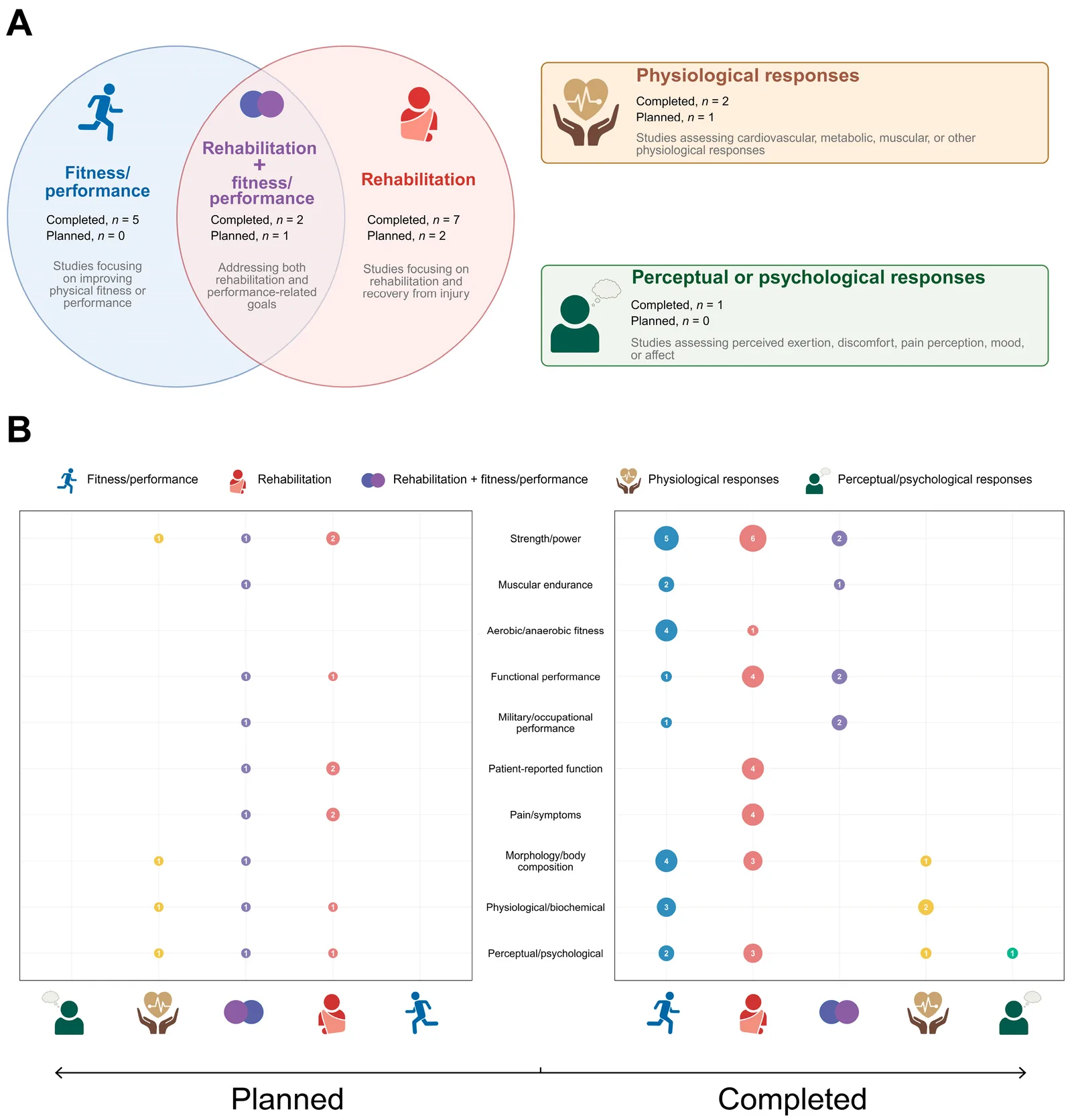
Application focus and outcome domains by evidence status. (**A**) Distribution of primary application focus in completed studies and protocol- or registry-only studies. (**B**) Distribution of planned and observed outcome domains across application-focus categories. Bubble size and labels indicate the number of studies. Individual studies could contribute to multiple outcome domains. Planned outcomes were derived from protocol- or registry-only studies and were not treated as observed evidence. Created with BioRender.com.

Application focus was summarized separately by evidence status (Figure 6A). Among the 17 completed studies, rehabilitation was the most common primary application focus (*n* = 7), followed by fitness/performance (*n* = 5), combined rehabilitation and fitness/performance (*n* = 2), physiological responses (*n* = 2), and perceptual or psychological responses (*n* = 1). Among the four protocol- or registry-only studies, two primarily focused on rehabilitation, one addressed both rehabilitation and fitness/performance, and one focused on physiological responses.

Observed outcomes reported in the 17 completed studies spanned multiple domains (Figure 6B). Strength/power was the most frequently assessed outcome domain (13/17, 76.5%), followed by morphology/body composition (8/17, 47.1%), functional performance (7/17, 41.2%), and perceptual/psychological outcomes (7/17, 41.2%). Muscular endurance and military/occupational performance were the least frequently assessed domains (3/17, 17.6% each). By application focus, rehabilitation-focused studies most commonly assessed strength/power (*n* = 6), functional performance (*n* = 4), patient-reported function (*n* = 4), and pain/symptoms (*n* = 4), whereas fitness/performance-focused studies most commonly assessed strength/power (*n* = 5), aerobic/anaerobic fitness (*n* = 4), and morphology/body composition (*n* = 4). Among the four protocol- or registry-only studies, strength/power was specified as a planned outcome in all four studies (4/4, 100%), followed by patient-reported function, pain/symptoms, physiological/biochemical outcomes, and perceptual/psychological outcomes (3/4, 75.0% each).

### 3.7. Safety and Implementation Characteristics

**Figure 7:**
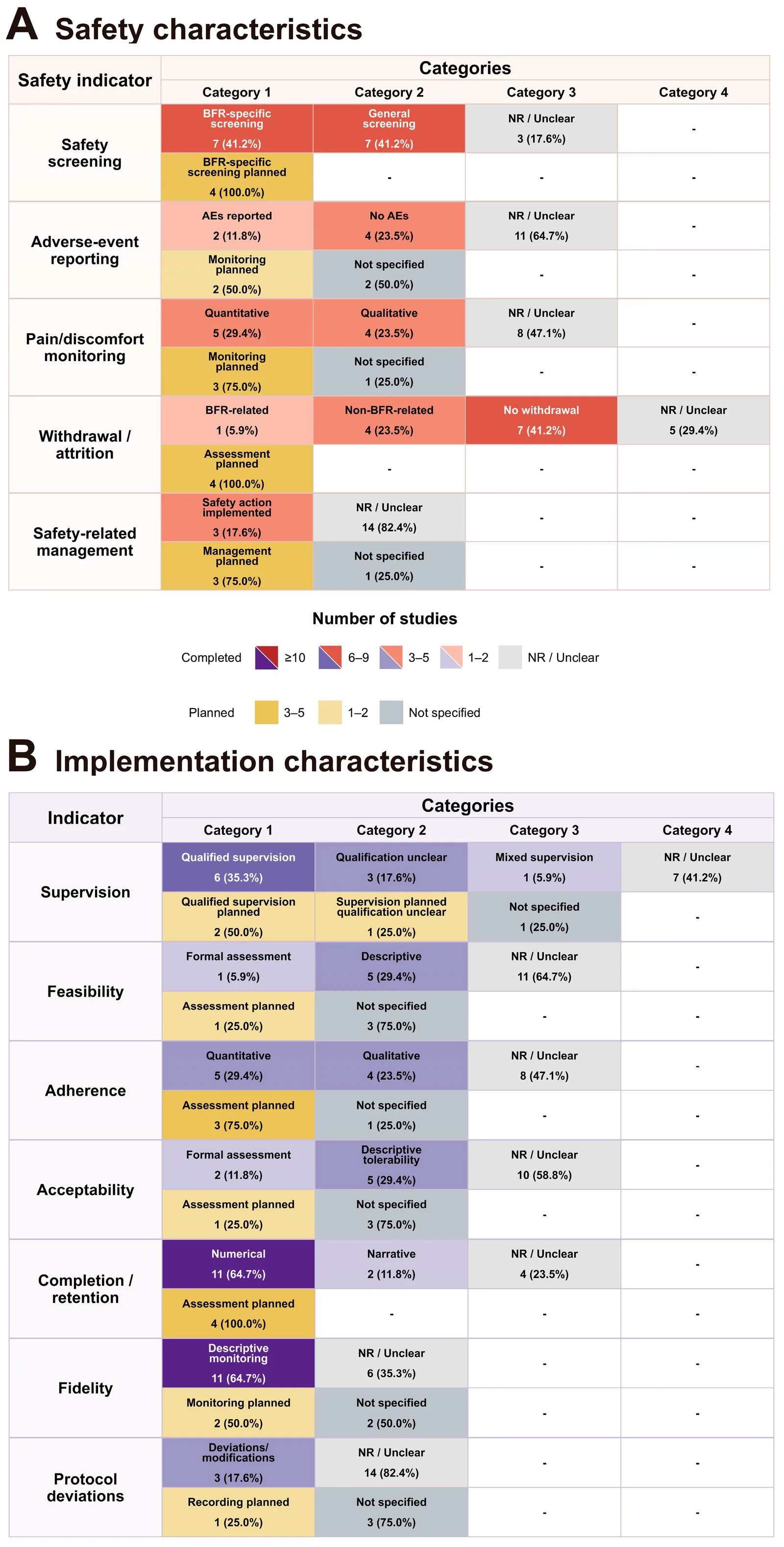
Safety and implementation characteristics by evidence status. (**A**) Distribution of safety-related reporting and monitoring characteristics. (**B**) Distribution of implementation characteristics. Counts and percentages were calculated separately for completed empirical studies (*n* = 17) and protocol- or registry-only studies (*n* = 4). For completed empirical studies, the categories represent reported safety and implementation procedures, monitoring practices, or observed outcomes. For protocol- or registry-only studies, the categories represent procedures or assessments explicitly planned in the protocol or registry record and should not be interpreted as observed implementation or outcomes. NR/Unclear indicates information that was not reported or could not be determined in completed empirical studies, whereas Not specified indicates information not specified in protocol- or registry-only studies. AE, adverse event; NR, not reported.

Among the 17 completed studies, 14 reported some form of safety screening, including seven that described BFRT-specific contraindication screening and seven that reported general health or eligibility screening. Three did not provide clear information on the screening approach. Adverse-event reporting was relatively incomplete. Two studies reported the occurrence of adverse events, whereas four explicitly reported no adverse events. The remaining 11 studies did not provide clear adverse-event information and were therefore classified as “not reported/unclear” rather than “no adverse events.” Pain or discomfort was quantitatively assessed in five studies and qualitatively described in four, whereas eight did not provide clear information. Regarding withdrawal or attrition, seven studies reported no participant withdrawals, four reported withdrawals for reasons unrelated to BFRT, one reported a BFRT- or symptom-related withdrawal, and five did not provide sufficient information. Three studies reported safety-related management actions or intervention modifications in response to symptoms or safety concerns, whereas the remaining 14 did not report or did not provide sufficient information to determine such actions. Among the four protocol- or registry-only studies, all planned BFRT-specific safety screening. Two planned adverse-event monitoring, three planned pain or discomfort monitoring, all four planned assessment or recording of withdrawal or attrition, and three specified planned safety-related management or response procedures (Figure 7A).

Implementation characteristics were similarly heterogeneous across the completed empirical studies. Six studies explicitly reported supervision by appropriately qualified professionals, three reported supervision without clearly identifying the supervisor’s qualifications, one used a combination of supervised and self-managed delivery, and seven did not report or did not provide sufficient information to determine the supervision approach. Feasibility was formally assessed in one study and addressed descriptively in five, whereas 11 did not provide clear feasibility information. Adherence was quantitatively reported in five studies and qualitatively reported in four, while eight did not provide clear information. Acceptability was formally assessed in two studies and described qualitatively in five, whereas 10 did not provide clear information. Completion or participant retention was reported more consistently, with 11 studies providing numerical information and two providing narrative information. Four did not provide clear information. Intervention fidelity was descriptively monitored in 11 studies, but no completed study formally quantified implementation fidelity, and six did not provide clear fidelity information. Three studies explicitly reported protocol deviations or implementation modifications, whereas the remaining 14 did not provide clear information. Among the four protocol- or registry-only studies, three planned supervision, including two that specified qualified professional supervision and one that did not clearly specify supervisor qualifications. One planned feasibility assessment, three planned to assess adherence, one planned acceptability assessment, all four planned assessment or recording of completion or retention, two planned intervention-fidelity monitoring, and one planned recording of protocol deviations (Figure 7B).

## 4. Discussion

This scoping review indicates that the number of completed empirical studies of BFRT in tactical settings has increased in recent years. The four protocol- or registry-only studies further indicate continued research activity in this area. However, the increase in completed studies has not been accompanied by a comparable increase in evidence maturity. Among the completed studies, the evidence base remains characterized by small samples, short intervention durations, and exploratory designs, with clear limitations in study populations, outcome selection, and the reporting of safety and implementation. Therefore, the available evidence warrants further investigation of BFRT in tactical physical fitness training and rehabilitation but remains insufficient to support its routine implementation in tactical populations. As additional completed evidence becomes available, greater attention should be given to its representativeness and translational relevance. Specifically, future research should determine whether existing findings are generalizable to the broader tactical workforce, how physiological and clinical outcomes relate to occupational task performance, and whether BFRT can be implemented safely and reproducibly in real-world tactical environments.

Although the target population of this review included multiple tactical occupational groups, such as military personnel, law enforcement officers, firefighters, and emergency medical personnel, all eligible studies identified in this review involved military populations. This indicates that the current evidence base for BFRT in tactical populations is substantially narrower than the range of occupations encompassed by this concept, and the findings cannot yet be directly generalized to other tactical occupations. Different tactical occupations may vary in task demands, injury profiles, age structure, and underlying health risks, and may therefore require different acceptable training loads, implementation conditions, and safety-screening procedures [3,52]. Similar concerns regarding representativeness also exist within military populations. Task demands, training loads, and injury risks are not uniform across different military subgroups [53]. However, existing studies often broadly describe participants as military personnel, with relatively limited reporting of specific service branches, occupational roles, and stages of service. In addition, although some studies included female participants, female-only samples are currently lacking and sex-stratified findings are rarely reported. Given that female military personnel may have distinct injury profiles and training needs [54,55,56], and that some studies suggest sex-related physiological differences may influence acute responses or long-term adaptations to BFRT [57,58], the applicability of the current evidence to military personnel of different sexes remains uncertain. Therefore, the generalizability of the identified evidence is limited. No eligible studies involving non-military tactical occupations were identified in this review, and representation across military service branches and sexes was also limited.

Existing BFRT research has examined training and rehabilitation contexts relevant to military personnel, but the occupational relevance of the current evidence remains limited. In terms of application context, BFRT has primarily been investigated along two pathways, injury rehabilitation and physical fitness enhancement, which broadly align with the practical needs of military personnel to maintain mission readiness and restore physical capacity after injury. Rehabilitation studies have mainly focused on lower-limb musculoskeletal conditions. This is consistent with evidence showing that lower-limb injuries are an important cause of work limitations and reduced mission readiness among military personnel [5,53]. In contrast, evidence for upper-limb rehabilitation remains limited, although impaired upper-limb function may also affect weapon and equipment handling, load carriage, and the performance of other military tasks [59,60]. Among healthy military personnel, BFRT has more commonly been used as an adjunct to physical fitness training. However, in both rehabilitation and fitness-enhancement settings, most assessed outcomes were indirect or surrogate measures of readiness, including strength, power, aerobic or anaerobic capacity, pain, and general physical function. Occupational performance outcomes were assessed in only 3 of the 17 completed studies. Although these indirect or surrogate outcomes are relevant to training and rehabilitation, they do not directly establish effects on occupational tasks such as loaded marching, casualty dragging, or equipment handling [61]. For military personnel recovering from injury or surgery, the goal of rehabilitation is not simply to restore function for activities of daily living, but to regain the capacity to perform physically demanding, complex, and potentially high-risk occupational tasks. Accordingly, direct readiness outcomes, such as return to duty and performance on occupation-specific or task-simulation tests, have greater occupational relevance. Future research should therefore more systematically incorporate occupational task-simulation tests, return-to-duty outcomes, and other direct measures of readiness.

Regarding BFRT prescription, several similarities were observed in commonly reported prescription components across the completed studies. Most studies used pneumatic cuffs and commonly prescribed relative cuff pressures based on individual AOP or LOP. Low-load resistance exercise combined with the traditional or modified 30–15–15–15 repetition scheme was also frequently used. However, substantial variation remains across studies in the actual level of applied pressure and cuff inflation–deflation patterns, and some studies incompletely reported key BFRT parameters. Systematic reviews of the broader BFRT literature have similarly identified considerable heterogeneity in pressure prescription and other prescription methods [62,63]. Such variation is not necessarily inappropriate, as clinical rehabilitation and physical fitness enhancement may require different BFRT prescriptions. The more important concern is that, when key BFRT parameters and the rationale for their selection are inadequately reported, it becomes difficult to determine whether between-study differences reflect appropriate adjustments for specific populations and research objectives or a lack of consistent methodological standards. Given that cuff pressure and inflation patterns may influence local physiological stimuli, exercise-related discomfort, and intervention tolerance [64], inadequate reporting further limits reproducibility, cross-study comparisons, and the assessment of dose–response relationships. In addition, most existing studies have used relatively short intervention periods without clearly explaining the rationale for their duration. Such designs are valuable for examining acute physiological responses or preliminary training adaptations. However, rehabilitation from military injuries and return to duty often involve substantially longer recovery periods [65]. It therefore remains unclear how strength and physical function change over longer follow-up periods, or whether responses to BFRT differ during later stages of recovery when higher training loads are reintroduced. Rather than simply evaluating a greater number of different BFRT protocols, future research should prioritize greater transparency in reporting BFRT prescriptions and the rationale underlying their selection. Studies targeting long-term training adaptations or injury rehabilitation should also adopt intervention and follow-up periods that are aligned with their research objectives and the expected recovery process. This would allow the persistence of adaptations, long-term tolerability, and the role of BFRT across the full course of training or rehabilitation to be evaluated.

Safety and implementation readiness are key issues that must be addressed before BFRT can move from controlled research settings into routine military and tactical practice. Among the completed studies, the reporting of safety-related information was inconsistent, with several safety-related domains frequently not reported or reported unclearly, particularly adverse events, pain or discomfort, withdrawals, and safety-related management procedures. Importantly, the absence of reporting should not be interpreted as indicating that safety-related procedures were not undertaken or that adverse events did not occur. Rather, incomplete reporting limits our ability to characterize these practices from the published evidence. Protocol- or registry-only studies described some planned safety procedures and monitoring, although the completeness with which these plans were specified varied across studies. Accordingly, the available evidence cannot be used to estimate the incidence of adverse events or to establish the safety of BFRT in tactical populations. Previous reviews in healthy and clinical populations have similarly identified inadequate monitoring and reporting of BFRT-related adverse events [66,67,68], but this limitation is particularly important in tactical settings. Military and tactical personnel may return relatively quickly to physically demanding or high-risk duties after rehabilitation, and symptoms, intolerance, or functional impairments that are not adequately identified during an intervention could affect subsequent task performance and personnel safety [69]. At the same time, the completed studies provide limited information on how BFRT was implemented in practice. Thus, the current uncertainty concerns not only whether BFRT is safe for individual participants, but also whether it can be delivered in a consistent and controlled manner. During deployments, field training, or operations in resource-limited environments, practical factors such as who delivers BFRT, what training providers require, whether equipment can operate reliably, whether the prescribed protocol can be implemented as intended, and how the protocol should be modified when abnormal symptoms or other safety concerns arise may be relevant to its practical implementation. Although these specific field-implementation factors were not directly evaluated in the included studies, they represent relevant considerations for future research on the translation of BFRT into operational settings. Future studies should therefore not only predefine and systematically monitor adverse events, but also evaluate feasibility, adherence, acceptability, and implementation fidelity. The completeness of intervention and safety reporting should also be improved by following established guidance such as TIDieR, CERT, and CONSORT Harms [70,71,72]. Only after both individual safety and real-world implementation conditions have been more comprehensively evaluated can it be determined whether BFRT is ready for broader adoption in routine tactical practice.

This scoping review has several limitations. First, no methodological quality or risk-of-bias assessment was conducted, and intervention effects were not quantitatively synthesized. Therefore, the review cannot determine the certainty of the evidence or estimate the overall effects of BFRT. Second, although the review protocol was finalized before the formal literature search, it was retrospectively registered on OSF after study screening and data extraction had been completed. Third, this review included both completed empirical studies and protocol- or registry-only studies. Although these two forms of evidence were distinguished throughout the synthesis, protocol- or registry-only studies provide information on intended rather than observed procedures and outcomes. Fourth, sex representation was characterized using study-level rather than participant-level counts. Therefore, the proportions of studies including male or female participants should not be interpreted as participant-level sex distributions. Fifth, backward and forward citation searching was not conducted, and the search strategy was not formally peer-reviewed by an information specialist. Consequently, potentially relevant poorly indexed studies or other grey literature may have been missed. Therefore, the absence of eligible studies involving non-military tactical populations should not be interpreted as evidence that such studies do not exist. Sixth, technical factors such as cuff width and material, device calibration, limb position, and the method used to determine occlusion pressure may influence BFRT delivery but were not systematically mapped in this review. Finally, two included reports [23,38] were primarily written in Persian. Machine-assisted translation was used during the review process and manually checked by the researchers, although minor translation or interpretive errors cannot be excluded.

## 5. Conclusions

The number of completed BFRT studies in tactical populations has increased, while planned research activity in this field is ongoing. Nevertheless, the completed evidence base remains at an early stage. All completed studies involved military personnel, and all protocol- or registry-only studies targeted military populations. No eligible studies involving other tactical occupations were identified. The completed evidence was characterized by small BFRT-exposed samples, short intervention durations, and limited assessment of direct readiness outcomes. BFRT has been investigated primarily in rehabilitation and training contexts, but the current evidence remains insufficient to support routine implementation or broad practice recommendations in tactical populations. Future research should include larger and longer studies, greater representation of women and non-military tactical populations, more direct assessment of occupationally relevant outcomes, and more systematic evaluation and reporting of BFRT safety, implementation, and prescription characteristics.

## Figures and Tables

**Table 1:** Inclusion and exclusion criteria based on the Population–Concept–Context (PCC) framework.

Framework	Inclusion Criteria	Exclusion Criteria
Population (P)	Tactical populations, including military service members, cadets/trainees, law enforcement officers, firefighters, emergency responders, paramedics, and other individuals explicitly identified as members of a military or tactical occupational population. Studies using broad labels such as “tactical athlete,” “tactical personnel,” or “tactical population” were eligible only when participants’ tactical occupational status could be clearly established from the available report.	Non-tactical populations; populations for whom tactical occupational status could not be established from the available report; and mixed populations for which eligible tactical subgroup data were not reported separately.
Concept (C)	Any form of blood flow restriction training or exercise, including BFRT combined with exercise-based rehabilitation or other exercise modalities.	Studies without BFRT, studies using occlusion only for surgical, diagnostic, passive vascular, or other non-exercise purposes, or records in which BFRT was not clearly linked to exercise or training.
Context (C)	Rehabilitation, pain management, return to duty, feasibility, training, performance, adherence, acceptability, or safety.	Studies unrelated to tactical training, rehabilitation, performance, or safety.

## Data Availability

No new data were created or analyzed in this study. Data sharing is not applicable to this article.

## Supplementary Materials

The following supporting information can be downloaded at https://www.mdpi.com/article/10.3390/healthcare14182920/s1, Table S1: PRISMA-ScR Checklist; File S1: Original Review Protocol; Table S2: Complete Search Strategies for All Databases and Trial Registries; Table S3: Study–Report Linkage, Data Extraction Sheet, and Coding Dictionary.

## Abbreviations

The following abbreviations are used in this manuscript:

1RM	One-repetition maximum
AOP	Arterial occlusion pressure
LOP	Limb occlusion pressure
BFRT	Blood flow restriction training
NMES	Neuromuscular electrical stimulation
NR	Not reported
PCC	Population–Concept–Context
RCT	Randomized controlled trial
SBP	Systolic blood pressure
AE	Adverse event
NS	Not specified
