# Supplementary material for: Blood Flow Restriction Training for Rehabilitation and Readiness in Tactical Populations: A Scoping Review
Source: Healthcare (Basel). 2026 Sep 9;14(18):2920. doi: 10.3390/healthcare14182920 (PMC13606564; doi:10.3390/healthcare14182920)
Supplement: Supplementary file 1 [file healthcare-14-02920-s001.zip › Supplementary File S1.pdf]

Original Review Protocol

Blood Flow Restriction Training for Rehabilitation and Readiness in Tactical Populations: A Scoping Review

1. Background

Tactical populations, including military personnel, law enforcement officers, firefighters, and emergency medical responders, require high levels of physical capacity and operational readiness but are also exposed to substantial training demands and musculoskeletal injury risk. Blood flow restriction training (BFRT) can provide meaningful physiological and neuromuscular stimuli at relatively low external loads, suggesting potential value for both physical training and injury rehabilitation in these populations. However, tactical personnel have distinct occupational demands and operational environments, and evidence from general or clinical populations may not be directly generalizable. Existing BFRT research in tactical populations remains fragmented, and its populations, applications, prescription characteristics, outcomes, safety, and implementation have not been comprehensively mapped. Therefore, a scoping review is warranted to systematically characterize the available evidence and identify key knowledge gaps.

2. Review Objective

This scoping review aims to systematically map the available evidence on BFRT in tactical populations, with a focus on study populations, applications, intervention characteristics, outcomes, safety, implementation, and key evidence gaps.

3. Review Questions

(1) Which tactical populations and BFRT applications have been studied?
(2) How are BFRT prescriptions and outcomes distributed?
(3) How are safety and implementation characteristics reported?

4. Eligibility Criteria

4.1 Population

Include:
Eligible populations will include tactical occupational populations, including:

- military service members;
- military cadets or trainees;
- law enforcement officers;
- firefighters;
- emergency responders;

- paramedics;
- other participants explicitly identified as members of a military or tactical occupational population.

Studies using broad terms such as “tactical athlete,” “tactical personnel,” or “tactical population” will be eligible only when the tactical occupational status of participants can be clearly established from the available report.

Exclude:

The following populations will be excluded:

- non-tactical populations;
- populations whose tactical occupational status cannot be established;
- mixed tactical and non-tactical samples when data relating specifically to the eligible tactical subgroup cannot be extracted separately.

4.2 Concept

Include:

Studies examining any form of blood flow restriction training or blood flow restriction exercise will be eligible, including BFRT combined with exercise-based rehabilitation or other exercise modalities.

Exclude:

Studies will be excluded when:

- BFRT is not involved;
- vascular or limb occlusion is used solely for surgical, diagnostic, or passive vascular purposes;
- occlusion is not clearly linked to exercise or training.

4.3 Context

Include:

Relevant contexts will include:

- Rehabilitation;
- pain management;
- return to duty;
- physical training;
- physical fitness or performance;
- feasibility;
- adherence;
- acceptability;
- safety.

No restriction will be imposed on the physical setting in which BFRT is delivered.

Exclude:

Studies unrelated to tactical training, rehabilitation, performance, or safety will be excluded.

5. Evidence Sources

Original studies, intervention studies, crossover studies, case reports/series, conference abstracts, study protocols, and clinical trial registrations with sufficient information will be included. Reviews, meta-analyses, editorials, and commentaries will be excluded. Multiple reports from the same study will be treated as one underlying study.

6. Information Sources

PubMed, Embase, Scopus, Web of Science, SPORTDiscus(EBSCO), CINAHL(EBSCO), Cochrane CENTRAL, ClinicalTrials.gov, and ISRCTN Registry.

7. Search Strategy

Search terms will be developed through discussion among the review authors and adapted to the controlled vocabulary, subject headings, free-text terms, field tags, and syntax requirements of each database.
The search will combine terms relating to blood flow restriction training with terms relating to tactical populations.
The overall conceptual search strategy will be:
("blood flow restriction" OR "blood-flow restriction" OR "blood flow restriction training" OR "blood flow restriction exercise" OR "blood flow restriction therapy" OR "BFR" OR "BFRT" OR "occlusion training" OR "vascular occlusion training" OR "KAATSU" OR "KAATSU training")
AND
("military" OR "soldier*" OR "servicemember*" OR "service member*" OR "warfighter*" OR "armed force*" OR "army" OR "navy" OR "naval" OR "marine*" OR "air force" OR "cadet*" OR "ROTC" OR "special force*" OR "special operation*" OR "tactical athlete*" OR "tactical population*" OR "tactical personnel" OR "tactical operator*" OR "police" OR "law enforcement" OR "firefighter*" OR "fire fighter*" OR "first responder*" OR "emergency responder*" OR "paramedic*" OR "emergency medical technician*" OR "EMT")
Database-specific search strategies will be adapted from this conceptual strategy.

8. Language

No language restrictions will be applied. Non-Chinese and non-English studies may be translated with machine-assisted tools and checked independently by two reviewers (Haonan Tian and Aozhe Wang).

9. Study Selection

Records will be managed in Zotero and duplicates removed. Two reviewers (Haonan Tian and Aozhe Wang) will independently screen titles/abstracts and full texts. For eligible trial registry records, related publications will be actively traced using registration numbers, study titles, investigator names, and intervention-related keywords. Registry records and corresponding publications will be linked and treated as multiple reports of the same

study. Disagreements will be resolved through discussion or adjudication by Jun Wang.

10. Data Extraction

Two reviewers will independently extract and cross-check data using a piloted electronic form. Missing information will be recorded as not reported or unclear.
Main extraction items will include:

- study year, country, and design;
- sample size and participant characteristics;
- tactical or military subgroup;
- health status and injury site;
- BFRT cuff and pressure prescription;
- exercise mode, intensity, structure, and duration;
- application focus and outcomes;
- safety reporting;
- feasibility, adherence, acceptability, supervision, fidelity, and protocol deviations.

11. Data Synthesis

Data will be summarized using descriptive statistics, narrative synthesis, tables, and graphical presentations.

12. Quality Assessment

No formal methodological quality assessment, risk-of-bias assessment, or meta-analysis will be conducted because the purpose is to map the evidence rather than estimate pooled intervention effects.
