# Supplementary material for: Blood Flow Restriction Training for Rehabilitation and Readiness in Tactical Populations: A Scoping Review
Source: Healthcare (Basel). 2026 Sep 9;14(18):2920. doi: 10.3390/healthcare14182920 (PMC13606564; doi:10.3390/healthcare14182920)
Supplement: Supplementary file 1 [file healthcare-14-02920-s001.zip › Supplementary Table S2.pdf]

Web of Science

```
TS=(
  ("blood flow" NEAR/2 restrict*)
  OR ("blood-flow" NEAR/2 restrict*)
  OR "blood flow restriction"
  OR "blood-flow restriction"
  OR BFR
  OR BFRT
  OR "occlusion training"
  OR "vascular occlusion training"
  OR KAATSU
)
AND
TS=(
  military
  OR soldier*
  OR servicemember*
  OR "service member*"
  OR warfighter*
  OR "armed force*"
  OR army
  OR navy
  OR naval
  OR "marine corps"
  OR marines
  OR "air force"
  OR cadet*
  OR ROTC
  OR "special force*"
  OR "special operation*"
  OR "special operations force*"
  OR "tactical athlete*"
  OR "tactical population*"
  OR "tactical personnel"
  OR "tactical operator*"
  OR police
  OR "law enforcement"
  OR firefighter*
  OR "fire fighter*"
  OR "first responder*"
```

OR "emergency responder*"
OR paramedic*
OR "emergency medical technician*"
OR EMT
)

PubMed

(
"Blood Flow Restriction Therapy"[Mesh]
OR "blood flow restriction"[tiab]
OR "blood-flow restriction"[tiab]
OR "blood flow restriction training"[tiab]
OR "blood flow restriction exercise"[tiab]
OR "blood flow restriction therapy"[tiab]
OR BFR[tiab]
OR BFRT[tiab]
OR "occlusion training"[tiab]
OR "vascular occlusion training"[tiab]
OR "KAATSU"[tiab]
OR "KAATSU training"[tiab]
)
AND
(
"Military Personnel"[Mesh]
OR "Military Health"[Mesh]
OR "Police"[Mesh]
OR "Firefighters"[Mesh]
OR "Emergency Responders"[Mesh]
OR "Emergency Medical Technicians"[Mesh]
OR military[tiab]
OR soldier*[tiab]
OR servicemember*[tiab]
OR "service member*"[tiab]
OR warfighter*[tiab]
OR "armed force*"[tiab]
OR army[tiab]
OR navy[tiab]
OR naval[tiab]
OR marine*[tiab]
OR "air force"[tiab]

```
  OR cadet*[tiab]
  OR ROTC[tiab]
  OR "special forces"[tiab]
  OR "special operations"[tiab]
  OR "tactical athlete*"[tiab]
  OR "tactical population*"[tiab]
  OR "tactical personnel"[tiab]
  OR "tactical operator*"[tiab]
  OR police[tiab]
  OR "law enforcement"[tiab]
  OR firefighter*[tiab]
  OR "fire fighter*"[tiab]
  OR "first responder*"[tiab]
  OR "emergency responder*"[tiab]
  OR paramedic*[tiab]
  OR "emergency medical technician*"[tiab]
  OR EMT[tiab]
)
```

SPORTDiscus

```
(
  TI (
    "blood flow restriction" OR "blood-flow restriction" OR
    "blood flow restriction training" OR "blood flow restriction exercise" OR
    "blood flow restriction therapy" OR
    BFR OR BFRT OR
    "occlusion training" OR "vascular occlusion training" OR
    KAATSU OR "KAATSU training"
  )
  OR AB (
    "blood flow restriction" OR "blood-flow restriction" OR
    "blood flow restriction training" OR "blood flow restriction exercise" OR
    "blood flow restriction therapy" OR
    BFR OR BFRT OR
    "occlusion training" OR "vascular occlusion training" OR
    KAATSU OR "KAATSU training"
  )
  OR SU (
    "blood flow restriction" OR "blood-flow restriction" OR
    "blood flow restriction training" OR "blood flow restriction exercise" OR
```

```
        "blood flow restriction therapy" OR
        BFR OR BFRT OR
        "occlusion training" OR "vascular occlusion training" OR
        KAATSU OR "KAATSU training"
    )
)
AND
(
    TI (
        military OR soldier* OR servicemember* OR "service member*" OR
        warfighter* OR "armed force*" OR army OR navy OR naval OR
        marine* OR "air force" OR cadet* OR ROTC OR
        "special forces" OR "special operations" OR
        "tactical athlete*" OR "tactical population*" OR
        "tactical personnel" OR "tactical operator*" OR
        police OR "law enforcement" OR firefighter* OR "fire fighter*" OR
        "first responder*" OR "emergency responder*" OR
        paramedic* OR "emergency medical technician*" OR EMT
    )
    OR AB (
        military OR soldier* OR servicemember* OR "service member*" OR
        warfighter* OR "armed force*" OR army OR navy OR naval OR
        marine* OR "air force" OR cadet* OR ROTC OR
        "special forces" OR "special operations" OR
        "tactical athlete*" OR "tactical population*" OR
        "tactical personnel" OR "tactical operator*" OR
        police OR "law enforcement" OR firefighter* OR "fire fighter*" OR
        "first responder*" OR "emergency responder*" OR
        paramedic* OR "emergency medical technician*" OR EMT
    )
    OR SU (
        military OR soldier* OR servicemember* OR "service member*" OR
        warfighter* OR "armed force*" OR army OR navy OR naval OR
        marine* OR "air force" OR cadet* OR ROTC OR
        "special forces" OR "special operations" OR
        "tactical athlete*" OR "tactical population*" OR
        "tactical personnel" OR "tactical operator*" OR
        police OR "law enforcement" OR firefighter* OR "fire fighter*" OR
        "first responder*" OR "emergency responder*" OR
        paramedic* OR "emergency medical technician*" OR EMT
    )
)
```

CINAHL

```
(
   MH "Blood Flow Restriction Therapy"
   OR TI (
      "blood flow restriction" OR "blood-flow restriction" OR
      "blood flow restriction training" OR "blood flow restriction exercise" OR
      "blood flow restriction therapy" OR
      BFR OR BFRT OR
      "occlusion training" OR "vascular occlusion training" OR
      KAATSU OR "KAATSU training"
   )
   OR AB (
      "blood flow restriction" OR "blood-flow restriction" OR
      "blood flow restriction training" OR "blood flow restriction exercise" OR
      "blood flow restriction therapy" OR
      BFR OR BFRT OR
      "occlusion training" OR "vascular occlusion training" OR
      KAATSU OR "KAATSU training"
   )
   OR SU (
      "blood flow restriction" OR "blood-flow restriction" OR
      "blood flow restriction training" OR "blood flow restriction exercise" OR
      "blood flow restriction therapy" OR
      BFR OR BFRT OR
      "occlusion training" OR "vascular occlusion training" OR
      KAATSU OR "KAATSU training"
   )
)
AND
(
   MH "Military Personnel"
   OR MH "Military Medicine"
   OR MH "Police"
   OR MH "Firefighters"
   OR MH "Emergency Medical Technicians"
   OR TI (
      military OR soldier* OR servicemember* OR "service member*" OR
      warfighter* OR "armed force*" OR army OR navy OR naval OR
      marine* OR "air force" OR cadet* OR ROTC OR
      "special forces" OR "special operations" OR
      "tactical athlete*" OR "tactical population*" OR
      "tactical personnel" OR "tactical operator*" OR
```

```
      police OR "law enforcement" OR firefighter* OR "fire fighter*" OR
      "first responder*" OR "emergency responder*" OR
      paramedic* OR "emergency medical technician*" OR EMT
   )
   OR AB (
      military OR soldier* OR servicemember* OR "service member*" OR
      warfighter* OR "armed force*" OR army OR navy OR naval OR
      marine* OR "air force" OR cadet* OR ROTC OR
      "special forces" OR "special operations" OR
      "tactical athlete*" OR "tactical population*" OR
      "tactical personnel" OR "tactical operator*" OR
      police OR "law enforcement" OR firefighter* OR "fire fighter*" OR
      "first responder*" OR "emergency responder*" OR
      paramedic* OR "emergency medical technician*" OR EMT
   )
   OR SU (
      military OR soldier* OR servicemember* OR "service member*" OR
      warfighter* OR "armed force*" OR army OR navy OR naval OR
      marine* OR "air force" OR cadet* OR ROTC OR
      "special forces" OR "special operations" OR
      "tactical athlete*" OR "tactical population*" OR
      "tactical personnel" OR "tactical operator*" OR
      police OR "law enforcement" OR firefighter* OR "fire fighter*" OR
      "first responder*" OR "emergency responder*" OR
      paramedic* OR "emergency medical technician*" OR EMT
   )
)
```

Embase

(
'blood flow restriction therapy'/exp
OR 'blood flow restriction':ti,ab,kw
OR 'blood-flow restriction':ti,ab,kw
OR 'blood flow restriction training':ti,ab,kw
OR 'blood flow restriction exercise':ti,ab,kw
OR 'blood flow restriction therapy':ti,ab,kw
OR BFR:ti,ab,kw
OR BFRT:ti,ab,kw
OR 'occlusion training':ti,ab,kw
OR 'vascular occlusion training':ti,ab,kw
OR 'vascular restriction training':ti,ab,kw
OR KAATSU:ti,ab,kw
OR 'KAATSU training':ti,ab,kw
)
AND
(
'military personnel'/exp
OR 'military medicine'/exp
OR 'police'/exp
OR 'firefighter'/exp
OR 'emergency responder'/exp
OR 'emergency medical technician'/exp
OR military:ti,ab,kw
OR soldier*:ti,ab,kw
OR servicemember*:ti,ab,kw
OR 'service member*':ti,ab,kw
OR warfighter*:ti,ab,kw
OR 'armed force*':ti,ab,kw
OR army:ti,ab,kw
OR navy:ti,ab,kw
OR naval:ti,ab,kw
OR marine*:ti,ab,kw
OR 'air force':ti,ab,kw
OR cadet*:ti,ab,kw
OR ROTC:ti,ab,kw
OR 'special forces':ti,ab,kw
OR 'special operations':ti,ab,kw
OR 'tactical athlete*':ti,ab,kw
OR 'tactical population*':ti,ab,kw
OR 'tactical personnel':ti,ab,kw

OR 'tactical operator*':ti,ab,kw
OR police:ti,ab,kw
OR 'law enforcement':ti,ab,kw
OR firefighter*:ti,ab,kw
OR 'fire fighter*':ti,ab,kw
OR 'first responder*':ti,ab,kw
OR 'emergency responder*':ti,ab,kw
OR paramedic*:ti,ab,kw
OR 'emergency medical technician*':ti,ab,kw
OR EMT:ti,ab,kw
)

Scopus

```
TITLE-ABS-KEY
(
   (
      "blood flow restriction"
      OR "blood-flow restriction"
      OR "blood flow restriction training"
      OR "blood flow restriction exercise"
      OR "blood flow restriction therapy"
      OR "BFR"
      OR "BFRT"
      OR "BFR training"
      OR "BFR exercise"
      OR "occlusion training"
      OR "vascular occlusion training"
      OR "vascular occlusion exercise"
      OR "KAATSU"
      OR "KAATSU training"
   )
   AND
   (
      "military"
      OR "military personnel"
      OR "military population*"
      OR "military service*"
      OR "service member*"
      OR servicemember*
      OR soldier*
```

OR warfighter*
OR "armed force*"
OR army
OR navy
OR naval
OR marine*
OR "air force"
OR cadet*
OR ROTC
OR "special force*"
OR "special operation*"
OR "special warfare"
OR "tactical athlete*"
OR "tactical population*"
OR "tactical personnel"
OR "tactical operator*"
OR police
OR "law enforcement"
OR firefighter*
OR "fire fighter*"
OR "first responder*"
OR "emergency responder*"
OR paramedic*
OR "emergency medical technician*"
OR EMT
)
)

Cochrane CENTRAL

#1 [mh "Blood Flow Restriction Therapy"]

#2 ("blood flow restriction" OR "blood-flow restriction" OR "blood flow restriction training" OR "blood flow restriction exercise" OR "blood flow restriction therapy"):ti,ab,kw

#3 (BFR OR BFRT):ti,ab,kw

#4 ("occlusion training" OR "vascular occlusion training" OR KAATSU OR "KAATSU training"):ti,ab,kw

#5 #1 OR #2 OR #3 OR #4

#6 [mh "Military Personnel"]

#7 [mh "Military Health"]

#8 [mh Police]

#9 [mh Firefighters]

#10 [mh "Emergency Responders"]

#11 [mh "Emergency Medical Technicians"]

#12 #6 OR #7 OR #8 OR #9 OR #10 OR #11

#13 (military OR soldier* OR servicemember* OR (service NEXT member*) OR warfighter* OR (armed NEXT force*) OR army OR navy OR naval OR marine* OR "air force" OR cadet* OR ROTC OR "special forces" OR "special operations"):ti,ab,kw

#14 ((tactical NEXT athlete*) OR (tactical NEXT population*) OR (tactical NEXT personnel) OR (tactical NEXT operator*)):ti,ab,kw

#15 (police OR (law NEXT enforcement) OR firefighter* OR (fire NEXT fighter*) OR (first NEXT responder*) OR (emergency NEXT responder*) OR paramedic* OR (emergency NEXT medical NEXT technician*) OR EMT):ti,ab,kw

#16 #12 OR #13 OR #14 OR #15

#17 #5 AND #16

ClinicalTrials.gov

("blood flow restriction" OR "blood-flow restriction" OR "blood flow restriction training" OR "blood flow restriction exercise" OR "blood flow restriction therapy" OR BFR OR BFRT OR "occlusion training" OR "vascular occlusion training" OR KAATSU OR "KAATSU training")
AND
(military OR soldier OR soldiers OR servicemember OR servicemembers OR "service member" OR "service members" OR warfighter OR warfighters OR "armed forces" OR army OR navy OR naval OR marine OR marines OR "air force" OR cadet OR cadets OR ROTC OR "special forces" OR "special operations" OR "special operations forces" OR "special warfare" OR "tactical athlete" OR "tactical athletes" OR "tactical population" OR "tactical populations" OR "tactical personnel" OR "tactical operator" OR "tactical operators" OR police OR "law enforcement" OR firefighter OR firefighters OR "fire fighter" OR "fire fighters" OR "first responder" OR "first responders" OR

"emergency responder" OR "emergency responders" OR paramedic OR paramedics OR "emergency medical technician" OR "emergency medical technicians" OR EMT OR EMTs)

ISRCTN Registry

1.
("blood flow restriction" OR BFR OR BFRT OR KAATSU) AND (military OR soldier OR soldiers OR army OR navy OR cadet OR cadets)

2.
("blood flow restriction" OR BFR OR BFRT OR KAATSU) AND ("tactical athlete" OR "tactical population" OR "tactical personnel")

3.
("blood flow restriction" OR BFR OR BFRT OR KAATSU) AND (police OR firefighter OR firefighters OR "first responder" OR paramedic)
